# Identifying gene clusters by discovering common intervals in indeterminate strings

**DOI:** 10.1186/1471-2164-15-S6-S2

**Published:** 2014-10-17

**Authors:** Daniel Doerr, Jens Stoye, Sebastian Böcker, Katharina Jahn

**Affiliations:** 1 Genome Informatics, Faculty of Technology, Bielefeld University, Bielefeld, Germany; 2 Institute for Bioinformatics, Center for Biotechnology (CeBiTec), Bielefeld University, Bielefeld, Germany; 3 Lehrstuhl für Bioinformatik, Friedrich-Schiller-Universität Jena, Jena, Germany; 4 Computational Biology Group, Department of Biosystems Science and Engineering, ETH Zürich, Basel, Switzerland

**Keywords:** common intervals, indeterminate strings, gene cluster detection

## Abstract

**Background:**

Comparative analyses of chromosomal gene orders are successfully used to predict gene clusters in bacterial and fungal genomes. Present models for detecting sets of co-localized genes in chromosomal sequences require prior knowledge of gene family assignments of genes in the dataset of interest. These families are often computationally predicted on the basis of sequence similarity or higher order features of gene products. Errors introduced in this process amplify in subsequent gene order analyses and thus may deteriorate gene cluster prediction.

**Results:**

In this work, we present a new dynamic model and efficient computational approaches for gene cluster prediction suitable in scenarios ranging from traditional gene family-based gene cluster prediction, via multiple conflicting gene family annotations, to gene family-free analysis, in which gene clusters are predicted solely on the basis of a pairwise similarity measure of the genes of different genomes. We evaluate our gene family-free model against a gene family-based model on a dataset of 93 bacterial genomes.

**Conclusions:**

Our model is able to detect gene clusters that would be also detected with well-established gene family-based approaches. Moreover, we show that it is able to detect conserved regions which are missed by gene family-based methods due to wrong or deficient gene family assignments.

## Background

Gene clusters are sets of functionally associated genes in prokaryotic and fungal genomes that are located close to each other over a longer period of evolutionary time, despite the genome undergoing significant rearrangements. Consequently, gene clusters may be found in several related species by means of comparative gene order analysis. Over the past years several such methods have been proposed and subsequently improved in their sensitivity. Initial gene cluster models considered only completely conserved genomic segments that retain gene order and orientation [1,2]. Later models still required gene clusters to be contiguous and complete but dropped the requirement for co-linearity [3-5]. The most powerful class of approaches can handle imperfect conservation of gene content by allowing to some extent either inserted [6-8] or both inserted and deleted genes [9-11].

All above methods require prior knowledge of homology relations between genes, using either a one-to-one mapping between the gene sets of different genomes [3,6,5], or a general partitioning into gene families [4,7-11]. In the latter, a genome is modeled as a set of sequences over the alphabet of gene families, where each sequence corresponds to a particular chromosome of the organism.

Most commonly, gene families are predicted computationally on the basis of sequence similarity. Various databases exist that offer information of precomputed gene families [12-14]. Furthermore, several software tools are freely available that allow for direct computation of gene family assignments in a dataset of interest [15-17]. Typically, these approaches assume that gene families naturally cluster into densely connected subgraphs in the gene similarity network. However, multi-domain proteins can have strong ties not only to their own family but also to other families they share a domain with. Some of these proteins may not be at all traceable back to a single gene family. While some recent approaches can deal with the ambiguities caused by multi-domain proteins [18,19], it is still a major challenge to define cut-offs in the clustering process that at the same time eliminate spurious edges and keep gene families at a reasonable granularity[20,21].

In this paper, we present a new dynamic model and efficient computational approaches for gene cluster prediction suitable in scenarios ranging from traditional gene family-based gene cluster prediction, via multiple conflicting gene family annotations, to gene family-free analysis, in which gene clusters are predicted solely on the basis of a pairwise similarity measure between the genes of different genomes. We do this by introducing the concept of *common intervals *to *indeterminate strings*, which are a class of strings that can have more than one character at every position. We then extend this concept to allow for a limited number of insertions and deletions. Furthermore, we present algorithms that solve related discovery problems of finding all *weak common intervals *and *approximate weak common intervals *in indeterminate strings. Finally, we propose a new method for gene family-free discovery of gene clusters based on (approximate) weak common intervals in indeterminate strings.

## Methods

### Definitions

Indeterminate strings, also known as *degenerate strings *are formally defined as [22]:

**Definition 1 (indeterminate string) ***For a given finite alphabet * ∑, *let *P(∑)*be the power set of * ∑*. An *indeterminate string *is a sequence of *character sets*, which are elements of *P(∑)\(∅).

In other words, for an indeterminate string *S *with *n *index positions must hold that for every *i*, 1≤i≤n, S[i]⊆∑ and S[i]≠∅, where S[i] denotes the character set associated with the *i*-th position in *S*. In the special case where every position of indeterminate string *S *holds a singleton set, *S *is equivalent to an ordinary string. We denote the *length *of an indeterminate string *S *with *n *index positions by |S|≡n and its *cardinality*, i.e. the number of *all *elements in *S*, by $∥S∥\equiv \sum_{i=1}^{n}|S\left[i\right]|$. Two positions *a *and *b*, 1≤a≤b≤|S|, induce the indeterminate *substring *S[a,b]≡S[a]S[a+1]…S[b]. To distinguish intervals in different indeterminate strings, we indicate the affiliation of an interval [i,j] to indeterminate string *S *by the subscript notation ${\left[i,j\right]}_{S}$.

**Example 1 **S={a,d,g}{c}{a,d}{e,f}{b}{c,g}*is an indeterminate string of length *|S|=6*and cardinality *||S||=11*over alphabet *∑={a,b,c,d,e,f,g}. *The third element of S is given by character set *S[3]={a,b}. *Interval *[2,4]*induces the substring *S[2,4]={c}{a,d}{e,f}.

In this work, we generalize the concept of common intervals, which were initially introduced on permutations [23] and subsequently extended to strings [24]. The idea behind common intervals is to compare strings, or rather substrings, based on their character sets. The character set of an ordinary string *S *is defined as C(S)≡{S[i] | 1≤i≤|S|}. The equivalent concept on indeterminate strings is the following:

**Definition 2 (character set) ***The *character set *of an indeterminate string  S is defined by *$C\left(S\right)\equiv {⋃}_{i=1}^{n}S\left[i\right]$.

In two ordinary strings *S *and *T *over a finite alphabet Σ, two intervals, [*i, j*] in *S *and [*k, l*] in *T*, are called *common intervals *if C(S[i,j])=C(T[k,l])). The analogon for indeterminate strings is:

**Definition 3 (strict common intervals) ***Given two indeterminate strings * S*and * T, *two intervals*, [i,j]*in * S*and *[k,l]*in * T, *are said to be *strict common intervals *if and only if their character sets *C(S[i,j])*and *C(T[k,l])*are equal*.

A weaker definition based on the intersection relation between character sets is:

**Definition 4 (weak common intervals) ***Given two indeterminate strings * S*and * T, *two intervals*, [i,j]*in * S*and *[k,l]*in * T, *are *weak common intervals *with *common character set C=C(S[i,j])∩C(T[k,l])*if for each * x, i≤x≤j, *it holds that *C∩S[x]≠∅*and for each * y, k≤y≤l, *it holds that *C∩T[y]≠∅.

In all our use cases, in particular when dealing with conflicting gene family assignments as well as gene family-free gene cluster detection, the concept of weak common intervals appears to be more appropriate. Thus, in the following, we restrict ourselves to the study of weak common intervals.

Furthermore, continuing a previous line of research initially proposed by Schmidt and Stoye in [4], we further extend weak common intervals by allowing a limited number of insertions and deletions:

**Definition 5 (approximate weak common intervals) ***Given two indeterminate strings * S*and * T*and a threshold *$\delta \in {\mathbb{N}}_{0}$, *two intervals*, [i,j]*in * S*and *[k,l]*in * T, *are *approximate weak common intervals *with *common character set C=C(S[i,j])∩C(T[k,l])*if the number of *positions *with no intersection with  C is limited by δ, i.e. |{x | i≤x≤j:S[x]∩C=∅}|+|{y | k≤y≤l:T[y]∩C=∅}|≤δ. These positions are called *indels.

Generally, algorithms for discovering common intervals of ordinary strings only report pairs of intervals that both are *maximal*, whereby *maximality *is defined as follows: An interval [i,j] in string *X *is called *maximal *if its immediate left and right neighboring characters, X[i-1] and X[j+1] (if such exist), are not contained in C(X[i,j]). In other words, interval [i,j] cannot be extended to its left or right without expanding the character set of the interval.

In terms of weak common intervals, we introduce the following property derived from [11]:

**Definition 6 ( C-closed) ***Given an indeterminate string * S, *an interval *[i,j], *and a character set *C⊆∑, *interval *[i,j]*is * C-closed *if *S[i], S[i]⊆C, *and if *i=1*or *S[i-1]∩C=∅, *and if *j=n*or *S[j+1]∩C=∅.

A reasonable balance between omitting expedient and including apparently redundant weak common intervals is found by the subset of those that are *mutually-closed*, as defined as follows:

**Definition 7 (mutually-closed) ***Given a pair of intervals *$({\left[i,j\right]}_{S},{\left[k,l\right]}_{T})$*of indeterminate strings * S and  T, $({\left[i,j\right]}_{S},{\left[k,l\right]}_{T})$*and *${\left[i,j\right]}_{S}$*are *mutually-closed *if *$({\left[i,j\right]}_{S},{\left[k,l\right]}_{T})$*is *C(T[k,l])-*closed and *${\left[i,j\right]}_{S}$C(S[i,j])-*closed*.

We consequently restrict the enumeration of weak common intervals and approximate weak common intervals to those that are mutually-closed.

*Combinatorial complexity*. The maximal number of mutually-closed weak common intervals of two indeterminate strings *S *and *T *of length *n *and *m*, respectively, is bounded by *nm*. This result follows from the fact that the number of intervals [*k, l*] in *T *that are mutually-closed weak common intervals with any interval with fixed left bound *i *in *S *is bounded by *m*. Likewise, the maximal number of mutually-closed approximate weak common intervals of indeterminate strings *S *and *T *is bounded by (*δ *+ 1)^2^*nm*.

*Gene family-free analysis*. In absence of gene family assignments, each gene in the dataset is represented by a unique *character*, linearly ordered along a *chromosomal string*. Therefore, the *n *characters of a chromosomal string can be identified by their integer *index set *{1, 2*, . . . , n*}. Relating characters of one chromosomal string to characters of another, we presume that we are given a symmetric *similarity measure σ_AB _*: *A *× *B → *ℝ_≥0 _for any two index sets *A *and *B*.

In gene family-free gene cluster analysis we aim at finding pairs of intervals in two chromosomal strings, whose characters are similar. We can reduce this problem to finding (approximate) weak common intervals between two indeterminate strings. To this end, we construct an *index mapping B_A_*:

$$B_{A}\left[y\right]=\left\{\begin{array}{cc} \left\{x\;|\;x\in A:\sigma_{AB}\left(x,y\right)>0\right\} & \text{if}\;\text{any}\;x\in A\;\text{exists}\;\text{s}\text{.t}\text{.}\;\sigma_{AB}\left(x,y\right)>0\\ \left\{\infty \right\} & \text{otherwise}. \end{array}\right)$$

Thus, *B_A _*is an indeterminate string over alphabet {1,2,…,|A|,∞}. Let $I_{A}=\left\{1\right\}\left\{2\right\}\cdots \left\{\left|A\right|\right\}$ represent the indeterminate string of *A*, a position in *I_A _*shares a character with a position in *B_A _*if and only if the similarity of the two corresponding characters is non-zero. Thus, finding intervals in chromosomal strings *A *and *B*, whose characters are similar, is equivalent to finding (approximate) weak common intervals of indeterminate strings *I_A _*and *B_A_*. Note that the set of (approximate) weak common intervals of *I_A _*and *B_A _*is identical to the one of *I_B _*and *A_B_*. The (approximate) weak common intervals differ in size and, most substantially, in the similarities between characters within the interval pairs. Therefore, we introduce a simple scoring scheme by which the solution space can be arranged into a landscape of highs and lows of (approximate) weak common intervals, ranked by taking into account the number and the similarities of the contained characters. We define a score function *µ*_xy _over an index *x *in index set *X *and an interval [*a, b*]*_Y _*in index set *Y *as

$$\mu_{XY}\left(x,\;{\left[a,b\right]}_{Y}\right)=\left\{\begin{array}{c} \frac{\underset{y\;\in \;{\left[a,\;b\right]}_{Y}}{\text{max}}\sigma_{\text{XY}}\left(x,y\right)}{\underset{z\;\in \;Y}{\text{max}}\sigma_{\text{XY}}\left(x,z)\right)}\;\;\text{if}\;\underset{z\;\in \;Y}{\text{max}}\sigma_{\text{XY}}\left(x,z\right)>0\\ 0\;\;\;\;\;\;\;\;\text{otherwise} \end{array}\right)$$

so that *µ*_xy _takes values between 0 and 1, being 1 if the gene with highest similarity to *x *lies within interval [*a, b*]*_Y_*. The overall score of two interval pairs ([*i, j*]*_A_*, [*k, l*]*_B_*) is then

$$score\left(\left({\left[i,j\right]}_{A},\;{\left[k,l\right]}_{B}\right)\right)=\overset{j}{\underset{x\;=\;i}{\sum }}\mu_{AB}\left(x,\;{\left[k,l\right]}_{B}\right)+\overset{l}{\underset{y\;=\;k}{\sum }}\mu_{BA}\left(y,{\left[i,j\right]}_{A}\right)$$

We now describe three algorithms to compute all mutually-closed weak common intervals and all mutually-closed approximate weak common intervals with at most *δ *indels in two indeterminate strings. Note that mutually-closed weak common intervals are a special subclass of mutually-closed approximate weak common intervals for *δ *= 0.

In the following, we consider two indeterminate strings *S *of length *n *and *T *of length *m*.

### Discovering weak common intervals

**Figure 1:**
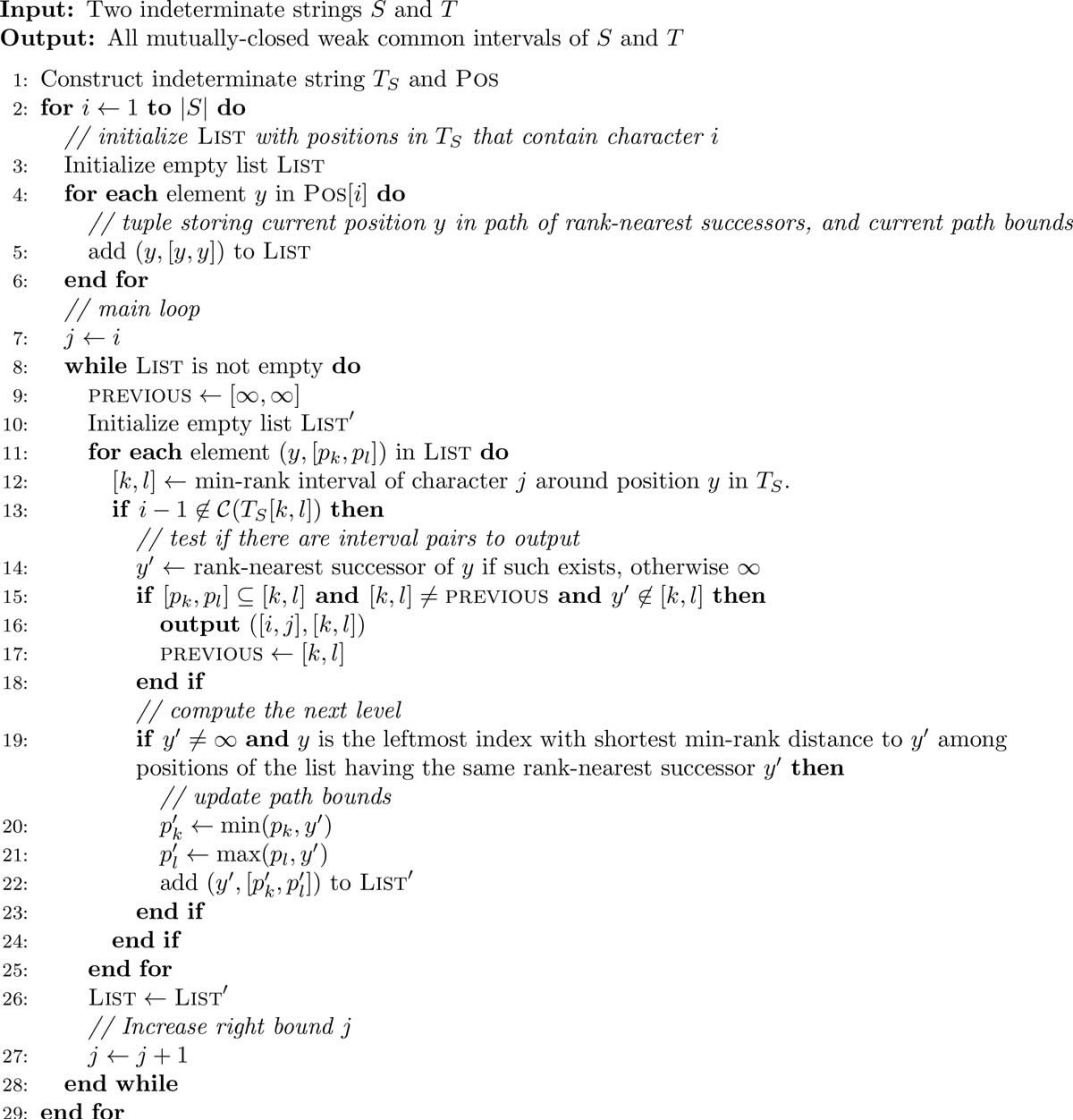
**WCII algorithm**. WCII adapts the search strategy of Didier's Algorithm [24] for common intervals in strings to the computation of weak common intervals in indeterminate strings.

We now describe the algorithm ***W**eak **C**ommon **I**ntervals on **I**ndeterminate **S**trings *(WCII) as presented in Figure 1. It solves the following problem:

**Problem 1 ***Given two indeterminate strings * S*and * T, *discover all mutually-closed weak common intervals of * S*and * T.

To tackle this problem we make use of the following constructs:

**Definition 8 (index string) ***Given an indeterminate string * S*of length * n, $I_{S}\equiv \left\{1\right\}\left\{2\right\}\cdots \left\{n\right\}$*denotes the *index string *of * S.

**Definition 9 (index mapping) ***Given two indeterminate strings * S*and * T*of lengths * n*and * m*respectively, the *index mapping *of * S*onto * T*is given by *${\left(T_{S}\left[y\right]\right)}_{y=1,\ldots ,m}$, *where*

$$T_{S}\left[y\right]=\left\{\begin{array}{cc} \left\{x\;|\;x=1,\;.\;.\;.\;,\;n:S\left[x\right]\cap T\left[y\right]\neq \emptyset \right\} & if\;T\left[y\right]\cap C\left(S\right)\neq \emptyset \\ \left\{\infty \right\} & otherwise. \end{array}\right)$$

Note that index strings and index mappings are again indeterminate strings. The key idea of WCII arises from the following observation:

**Observation 1 ***Given two indeterminate strings * S*and * T*with index string *$I_{S}$*and index mapping *$T_{S}$, *two intervals *[i,j]*in * S*and *[k,l]*in * T*are weak common intervals if and only if *${\left[i,j\right]}_{I_{S}}$*and *${\left[k,l\right]}_{T_{S}}$*are weak common intervals*.

This equivalence holds because any two positions, *x *in *S *and *y *in *T *intersect if and only if *I_S_*[*x*] and *T_S_*[*y*] intersect. Since it holds that *I_S_*[*x*] = {*x*} for all *x *= 1*, . . . , n*, we simplify the notation of single character set *I_S_*[*x*] to just *x *and character set $C\left(I_{S}\left[i,j\right]\right)$ to just [i,j]. Note that character $c\in C\left(I_{S}\left[i,j\right]\right)$ serves subsequently both as character *c *ϵ [*i, j*] as well as index in *I_S_*.

WCII is an adaptation of Didier's Algorithm [24] of enumerating maximal common intervals in ordinary strings. Didier's strategy can be described as follows: The algorithm iterates over all positions *i *in *S *as possible left interval bounds. In each iteration all mutually-closed weak common interval pairs are reported that share the same left bound *i *in *I_S_*. For each possible right bound *j *≥ *i*, the algorithm iterates through the set of positions in *T_S _*that contain *j *in their character set. To this end, we make use of an array POS, where POS[*j*], 1 ≤ *j *≤ *n*, is a sorted list of positions in *T_S _*containing character *j*. Each position *y *ϵ POS[*j*] is associated with an interval ${\left[k,l\right]}_{T_{S}}$, *k *≤ *y *≤ *l*, called the *min-rank interval *of character *j *for position *y*. It is the largest interval around *y *for which every position in ${\left[k,l\right]}_{T_{S}}$ contains at least one character in [*i, j*]. Obviously, ${\left[k,l\right]}_{T_{S}}$ is [*i, j*]-closed. It remains to be checked if ${\left[i,j\right]}_{I_{S}}$ is closed w.r.t. $C\left(T_{S}\left[k,l\right]\right)$ and that every position in ${\left[i,j\right]}_{I_{S}}$ and ${\left[k,l\right]}_{T_{S}}$ contains a character from $C=C\left(I_{S}\left[i,j\right]\right)\cap C\left(T_{S}\left[k,l\right]\right)$. To show the latter, it is sufficient to show that $\left[i,j\right]\subseteq C\left(T_{S}\left[k,l\right]\right)$, because the character set of each position in *I_S _*corresponds to the single element set of its index. The details of both tests are explained below, after relevant data structures are introduced. If both conditions are satisfied, a mutually-closed weak common interval pair is found and subsequently reported.

Like in Didier's Algorithm, we employ two tricks that improve the performance: precomputing *min-rank intervals *and following paths of *rank-nearest successors*.

*Precomputing min-rank intervals*. In order to identify min-rank intervals, it is sufficient to observe the smallest character *c *≥ *i *in each position. To this end, we make use of the following construct:

**Definition 10 (*i*-reduced string) ***Given index mapping *$T_{S}$, ${\left(T_{S}^{i}\left[y\right]\right)}_{y\;=\;1,...,m}$*is the * i-reduced string of $T_{S}$ of the *i*th iteration, *where *$T_{S}^{i}\left[y\right]=\text{min}\left(\left\{c\;|\;c\in T_{S}\left[y\right]\cup \left\{\infty \right\}:c\geq i\right\}\right)$.

Min-rank intervals in $T_{S}^{i}$ are identical to *rank intervals *as initially defined by Dider *et al*. [24]. Interestingly, rank intervals in $T_{S}^{i}$ correspond directly to min-rank intervals in *T_S_*:

**Lemma 1 ***The set of min-rank intervals in *$T_{S}$*is identical to the set of rank intervals in *$T_{S}^{i}$.

*Proof: *Didier *et al*.[24] show that rank intervals in a string are nested and that their number is bounded by the length of the string.

Observe that for any position *y *in $T_{S}^{i}$ the rank interval of character $j=T_{S}^{i}\left[y\right]$ is identical to the min-rank interval of *j *at position *y *in *T_S_*. Let *y *be a position in *T_S _*and *j *ϵ *T_S_*[*y*] such that $j>T_{S}^{i}\left[y\right]$. Further, let ${\left[k,l\right]}_{T_{S}}$ be the min-rank interval of *j *at *T_S_*[*y*], $j\prime =\text{max}\left(\left\{c\;|\;c\in C\left(T_{S}^{i}\left[k,l\right]\right):c\leq j\right\}\right)$, and ${\left[k\prime ,l\prime \right]}_{T_{S}}$ be the min-rank interval of *j*' at its corresponding position in *T_S_*. Because *j*' ≤ *j *it consequently holds that ${\left[k\prime ,l\prime \right]}_{T_{S}}\subseteq {\left[k,l\right]}_{T_{S}}$. Now, according to the definition of min-rank intervals, $T_{S}^{i}\left[k\prime -1\right]>j\prime$, if such position exists. Since *j*', is the largest character in $T_{S}^{i}\left[k,\;l\right]$ that is smaller than or equal to *j*, it must also hold that $T_{S}^{i}\left[k\prime -1\right]>j$. The same argument holds for $T_{S}^{i}\left[l\prime +1\right]$ if such position exists, therefore ${\left[k,l\right]}_{T_{S}}={\left[k\prime ,l\prime \right]}_{T_{S}}$ is the min-rank interval of both characters *j*' and *j*. We conclude that all min-rank intervals for any character in *T_S _*at iteration *i *are contained in the set of rank intervals of $T_{S}^{i}$.   □

Consequently, all min-rank intervals in *T_S _*in the *i*th iteration (i.e. for a fixed left bound *i *in *I_S _*) can be precomputed in O(m) time using the algorithm given by Didier *et al*. [24]. They are stored in table INT. For a currently processed character *j *at position *y *in *T_S_*, INT[*y*] contains its corresponding min-rank interval. Unlike Didier's Algorithm, INT must be updated after each iteration such that all positions in INT accessed in the following (*j *+ 1)th iteration contain the corresponding min-rank intervals of character *j *+ 1. Details of the update step can be found in Additional file 1 Section 1.1.

*Following paths of rank-nearest successors*. The second trick in the algorithm consists in increasing the right bound *j *in *I_S _*while walking through positions and characters of *T_S_*. Thereby the algorithm jumps from a current position *y *that contains character *j *to its *rank-nearest successor*, which is the position *y*' containing character *j *+ 1 with the smallest *min-rank distance *to *y *as defined as follows:

**Definition 11 (min-rank distance) ***The *min-rank distance *of any two positions  k and  l in indeterminate string $T_{S}$ for the ith iteration is given by:*

$$d_{T_{S}}^{i}\left(k,l\right)\equiv \text{max}\left(\{T_{S}^{i}\left[p\right]|k\leq p\leq l\right\})$$

If several co-optimal positions are available, the tie is broken by choosing the leftmost one as rank-nearest successor. In case no position with character *j *+ 1 exists, or the smallest min-rank distance is '*∞*', *j *has no successor. For the *i*th iteration, all rank-nearest successors are precomputed and stored in table SUCC which is explained in more detail in Additional file 1 Section 1.2.

Connecting characters larger than or equal to *i *at their corresponding positions in *T_S _*with their rank-nearest successors through directed edges results in a forest of rooted trees. Nodes (across all trees) sharing the same character are said to reside on the same *level*. In lines 8-28 of Figure 1, the algorithm traverses along paths through this forest in a bottom-up procedure, from one level to the next, starting at those leaves with character *i*. Besides the currently visited nodes of the level, the algorithm keeps track of the *path bounds*, which are the outermost positions in *T_S _*a path has visited thus far. The currently visited nodes of the paths and their corresponding path bounds are stored in a list labeled LIST. Only after all nodes of the same level *j *are processed, the algorithm follows all current paths to nodes of the next level *j *+ 1, thereby ensuring that each character in *T_S _*is processed at most once. To this end, for all positions containing character *j *that have the same rank-nearest successor *y*', the algorithm discontinues the paths of all but the leftmost one with shortest min-rank distance to *y*' (line 19). Traversing along paths of rank-nearest successors in WCII differs from Didier's Algorithm by the fact that a position in *T_S _*may be visited by the same path several times on different levels.

For any given min-rank interval ${\left[k,l\right]}_{T_{S}}$ there cannot be more than one weak common interval partner in *I_S _*starting at position *i*. Therefore it is sufficient to track at least one path in each min-rank interval to find all mutually-maximal intervals of *I_S _*and *T_S_*. Positions in POS are sorted, thus paths leading to the same weak common interval pair appear adjacent to each other in LIST and the common interval pair is reported only for the first (lines 15-17).

For each node in LIST, associated with character *j *and position *y*, the algorithm checks if the min-rank interval ${\left[k,l\right]}_{T_{S}}$ of *j *encloses the path bounds up to position *y *(see condition in line 15). If validated, a weak common interval pair has been found, given by ${\left([i,j\right]}_{I_{S}},\;{\left[k,l\right]}_{T_{S}})$. To ensure mutual closedness, the interval pair is only reported if *i − *1 is not contained in the character set $C\left(T_{S}|k,l|\right)$ and the successor of *y *is not within the current bounds of its path (see conditions in lines 13 and 15). Checking for the former can be achieved in O(1) time after O(m) time preprocessing by performing a range minimum query on an array of size O(m) where each position containing character *i − *1 is assigned 0 and 1 otherwise.

The overall complexity of the algorithm can be summarized as follows: Each position in *I_S _*is regarded exactly once as left bound *i *for all weak common intervals that are reported in one iteration. Once $T_{S}^{i}$ is computed for *i *= 1 it can be up-dated using array POS, taking overall $O\left(\left||T_{S}|\right|\right)$ time for all left bounds *i *= 1*, . . . , n*. Further, for each left bound the algorithm performs O(m) steps to precompute all min-rank intervals and $O\left(\left||T_{S}|\right|\right)$ steps to precompute all rank-nearest successors. The subsequent bottom-up procedure and the reporting of weak common intervals requires again $O\left(\left||T_{S}|\right|\right)$ time. Therefore we have:

**Theorem 1 ***Given two indeterminate strings * S*and * T, *Algorithm WCII finds all pairs of mutually-closed weak common intervals of * S*and * T*in *$O\left(n||T_{S}||\right)$ time.

### Discovering approximate weak common intervals

**Figure 2:**
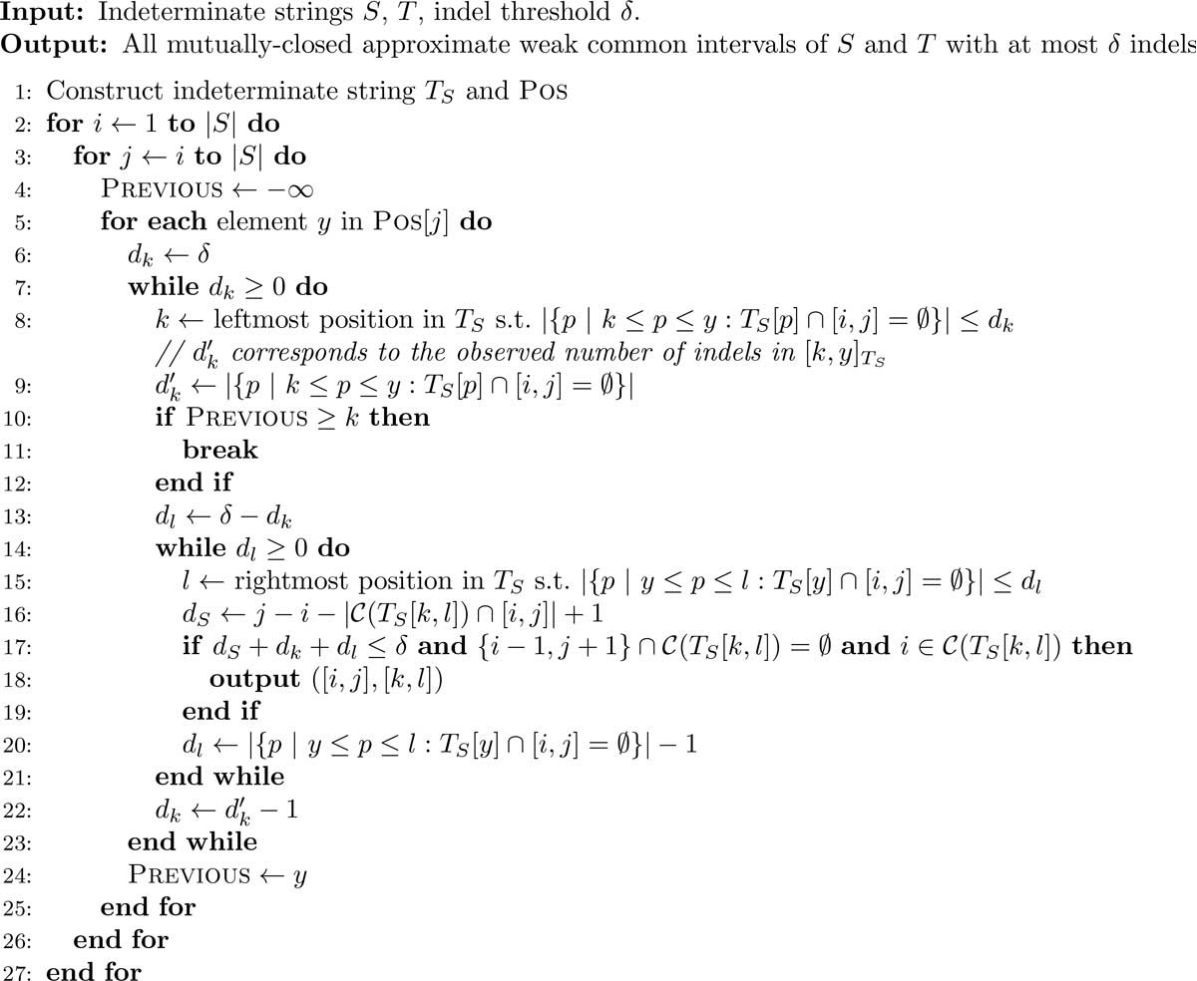
**AWCII algorithm**. AWCII is a search algorithm for approximate weak common intervals in indeterminate strings. It is an adaptation of RGC [11], an algorithm for computing approximate common intervals in strings.

We now present the algorithm ***A**pproximate **W**eak **C**ommon **I**ntervals on **I**ndeterminate **S**trings *(AWCII) as presented in Figure 2, thus line numbers mentioned in this subsection refer to Figure 2. AWCII solves the following problem:

**Problem 2 ***Given two indeterminate strings * S*and * T*and indel threshold *$\delta \in {\mathbb{N}}_{0}$, *discover all mutually-closed approximate weak common intervals of * S*and * T*with no more than * δ indels.

Following a strategy similar to WCII, AWCII solves Problem 2 for index mappings *I_S _*and *T_S_*, instead of *S *and *T*. As before, in each iteration the algorithm maintains a fixed left bound *i *in *I_S_*. For each character *j *ϵ [*i, n*] all positions *y *in *T_S _*are processed that contain character *j *(lines 5-25). Thereby character *j *at position *y *in *T_S _*can be associated with several different intervals around *y *that are candidates of mutually-closed approximate weak common interval partners for interval ${\left[i,j\right]}_{I_{S}}$. Only intervals surrounding one (or several) positions *y *can be mutually-closed. However, for an interval ${\left[k,l\right]}_{T_{S}}$ containing indels, it no longer holds that the minrank distance of any two positions within the interval is always smaller than the min-rank distance from any position inside to any position outside the interval. As a result, neither precomputed min-rank intervals nor following paths of ranknearest successors can be used for improving the algorithm's performance. Instead we pursue a different approach, thereby making AWCII an adaptation of the RGC algorithm of Jahn [11].

For each *d_k _*= 1*,..., δ *(lines 7-23) AWCII identifies the leftmost position *k *in *T_S _*such that at most *d_k _*indels are contained in interval ${\left[k,y\right]}_{T_{S}}$ and *T_S_*[*k*] ∩ [*i, j*] ≠ ∅. Let ${d\prime }_{k}\leq d_{k}$ be the observed number of indels in ${\left[k,l\right]}_{T_{S}}$ (see line 9), the algorithm then finds for each *d_l _*= 1*,..., δ − d*'*_k _*(lines 14-21) the rightmost position *l *such that again *T_S_*[*l*] ∩ [*i, j*] ≠ ∅ and the number of indels in ${\left[y,l\right]}_{T_{S}}$ does not exceed *d_l_*. Each (*k, l*) of the at most (*δ *+ 1)^2 ^combinations of leftmost and rightmost positions gives rise to a candidate pair of mutually-closed approximate weak common intervals ${(\left[i,j\right]}_{I_{S}},\;{\left[k,l\right]}_{T_{S}})$. For each candidate pair it is checked that the number of characters in [*i, j*] not contained in $C\left(T_{S}\left[k,l\right]\right)$ plus the already consumed number of indels in ${\left[k,l\right]}_{T_{S}}$ does not exceed *δ*. Finally, it is tested if ${\left[i,j\right]}_{I_{S}}$ is $C\left(T_{S}\left[k,l\right]\right)$-closed. If both conditions are satisfied, a mutually-closed approximate weak common interval pair is found and is subsequently reported (line 18).

Runtime improvements are achieved by precomputing right and left bounds of candidate intervals ${\left[k,l\right]}_{T_{S}}$ for each character *j *in *T_S_*. These bounds are computed within $O\left(\left(\delta +1\right)||T_{S}||\right)$ time for a fixed left bound *i *in *I_S _*and stored in tables L and R respectively. Further, for each such candidate interval ${\left[k,l\right]}_{T_{S}}$ the number of characters that are within [*i, j*] can also be precomputed. This number is used to determine *δ_S _*in line 16. The construction of the corresponding table, called RANGECONTENT, is achieved within $O\left({\left(\delta +1\right)}^{2}n||T_{S}||\right)$ time for a fixed left bound *i*. The details of constructing tables L, R, and RANGECONTENT can be found in Additional file 1 Section 2. Note that RANGECONTENT differs significantly from the data structure NUM used in RGC to count characters in intervals.

In terms of overall runtime, for each fixed bound *i *in *I_S _*the algorithm spends $O\left({\left(\delta +1\right)}^{2}n||T_{S}||\right)$ time on computation of the above mentioned auxiliary tables. Thereafter, AWCII requires $O\left({\left(\delta +1\right)}^{2}||T_{S}||\right)$ time to iterate through all combinations of candidate intervals in L and R and to identify approximate weak common intervals.

Testing for $C\left(T_{S}\left[k,l\right]\right)$-closedness of interval ${\left[i,j\right]}_{I_{S}}$ can be achieved in O(1) time by precomputing a table for all candidate intervals in *T_S _*of the *i*th iteration, where each entry indicates if a character *i − *1 or *j *+ 1 is contained in the corresponding candidate interval. Such a table can be constructed within $O\left(\left(\delta +1\right)\cdot ||T_{S}||\right)$ time using again a simple sweep algorithm. We conclude with the following theorem:

**Theorem 2 ***Given two indeterminate strings * S*and * T*and indel threshold *$\delta \in {\mathbb{N}}_{0}$, *algorithm AWCII computes all pairs of mutually-closed approximate weak common intervals of * S*and * T*in *$O\left({\left(\delta +1\right)}^{2}\cdot n^{2}||T_{S}||\right)$*time*.

### A runtime heuristic for discovering approximate weak common intervals

**Figure 3:**
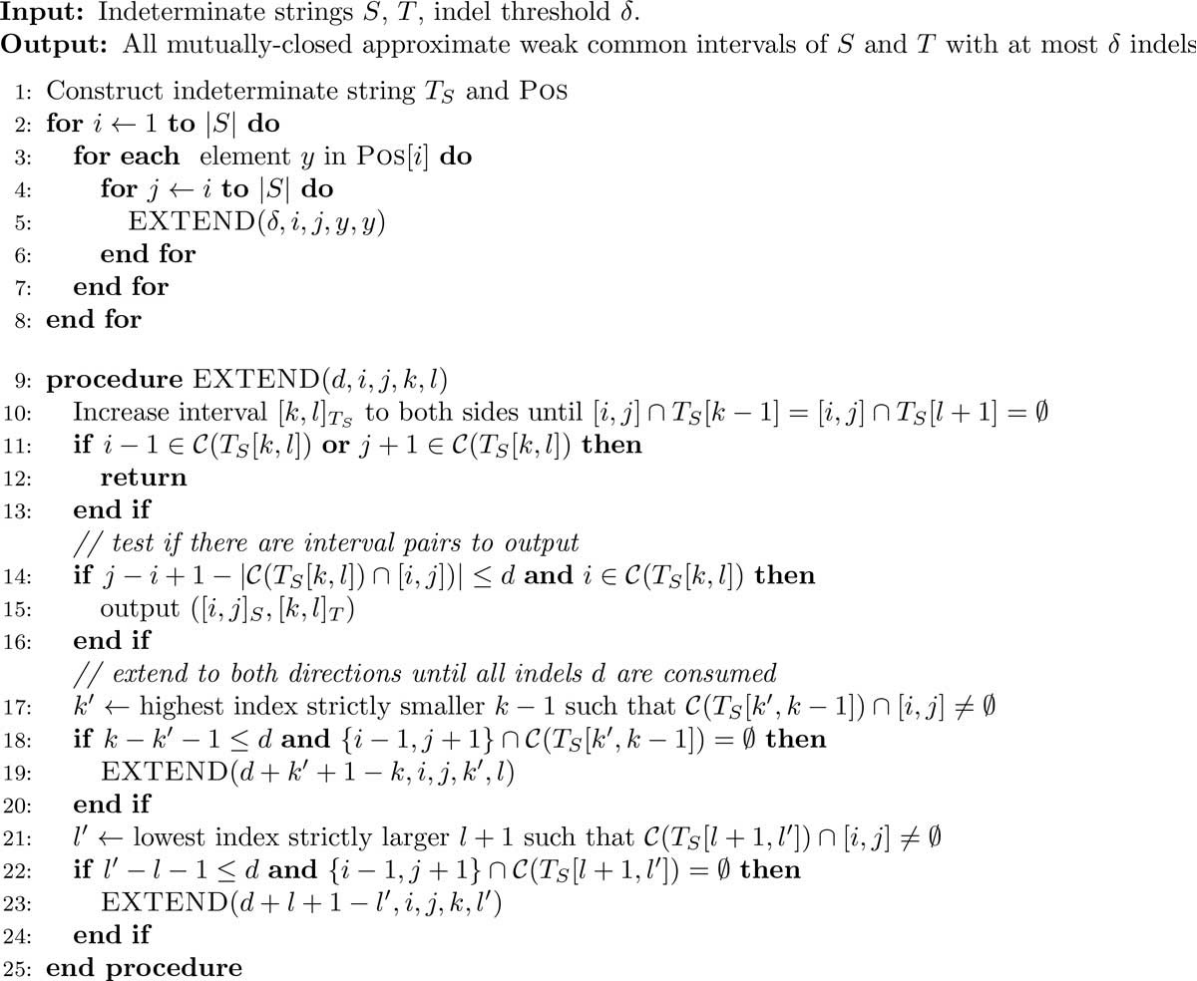
**ACSI algorithm**. ACSI is a runtime heuristic that computes all approximate weak common intervals in indeterminate strings.

Our third algorithm, ACSI (see Figure 3) represents a runtime heuristic that solves Problem 2 exactly and in practice outperforms both WCII and AWCII in gene family-free analysis by orders of magnitude.

Just as the two algorithms before, ACSI operates on index string *I_S _*and index mapping *T_S _*instead of indeterminate strings *S *and *T *directly. For every fixed interval [*i, j*] in *I_S_*, ACSI identifies mutually-closed approximate weak common interval partners [*k, l*] in *T_S_*. To this end, it iterates through elements of POS[*i*], i.e. positions in *T_S _*that contain character *i *(lines 3-7 of Figure 3). For each such position *y *ϵ POS[*i*] the algorithm calls a recursive procedure, denoted EXTEND (line 5). This recursive procedure requires 5 parameters, corresponding to fixed bounds ${\left[i,j\right]}_{I_{S}}$, the currently processed interval [*k, l*] in *T_S_*, and the current number of allowed indels, *d*. In the initial call, interval ${\left[k,l\right]}_{T_{S}}$ is set to ${\left[y,y\right]}_{T_{S}}$ and *d *= *δ*. EXTEND then increases interval ${\left[k,l\right]}_{T_{S}}$ to both sides until [*i, j*] ∩ *T_S_*[*k − *1] = ∅ and [*i, j*] ∩ *T_S_*[*l *+ 1] = ∅ (line 10). If in this process the algorithm observes characters *i − *1 or *j *+ 1 in $C\left(T_{S}\left[k,l\right]\right)$, EXTEND returns to the previous call (lines 11-13). Otherwise, it verifies if ${(\left[i,j\right]}_{I_{S}},\;{\left[k,l\right]}_{T_{S}})$ is a mutually-closed approximate weak common interval pair by testing if the number of characters in [*i, j*] that are missing in $C\left(T_{S}\left[k,l\right]\right)$ is less than or equal to the current *d *and if $i\in C\left(T_{S}\left[k,l\right]\right)$ (line 14). The interval pair is reported if both conditions are validated. EXTEND then increases ${\left[k,l\right]}_{T_{S}}$ to the left, thereby consuming indel positions as long as their overall number remains less than or equal to the current *d *(line 17). If a position *k*' *< k − *1 has been found such that [*i, j*] ∩ *T*[*k*'] ≠ ∅, EXTEND is called recursively with parameter values ${\left[i,j\right]}_{I_{S}}$, ${\left[k\prime ,l\right]}_{T_{S}}$, and the remaining number of allowed indels, given by *d *+ *k*' + 1 *− k *(lines 18-20). This step is also symmetrically executed for the right side of ${\left[k,l\right]}_{T_{S}}$ (lines 21-24).

The actual heuristic speed-up of the algorithm is achieved by calling procedure EXTEND in line 5 not for all intervals [*i, j*] in *I_S _*but only for those that have chances of success for being a weak common intervals pair with an interval [*k, l*] around a position *y *ϵ POS[*i*]. Thus, the neighborhood around position *y *is scanned for suitable values of *j *prior to the execution of EXTEND. The details are described in Additional file 1 Section 3.

## Results and discussion

In the following, we highlight the benefit of our dynamic model in comparison with present approaches. Although conflicting gene family assignments are extremely common in practice, this scenario is difficult to evaluate. Assuming the existence of an ultimately true gene family assignment, conflicts arise by incorrect gene family assignments. Therefore an evaluation would inevitably result in benchmarking gene family prediction tools, rather than scrutinizing our model.

Instead, we decided to evaluate our gene family-free model against the traditional gene family-based approach. To this end, we chose a genomic dataset of bacterial genomes that has been used in a prior gene cluster study [8] and was originally obtained from [25]. The dataset features 133 chromosomal sequences, of which we removed all sequences originating from plasmids.

**Figure 4:**
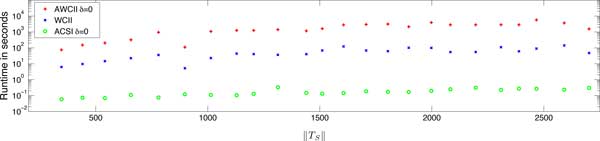
**Runtimes of presented algorithms in practice**. Running times of ACSI and AWCII with *δ *= 0 and WCII, measured in a sample of 24 arbitrarily chosen pairwise comparisons of genomes that are contained in the studied dataset. All algorithms produced identical output (as expected). Running times are plotted against the number of pairwise gene similarities (equivalent to the size of $||T_{S}||$) contained in the pairwise comparison.

In practice ACSI outperforms both WCII and AWCII as shown by Figure 4. Thus, in all subsequent results, we used ACSI to compute mutually-closed (approximate) weak common intervals.

*Gene family-based dataset*. Genes in this dataset are annotated with COG (Clusters of Orthologous Groups) identifiers [12] which are used to establish homology relationships between genes. The set of genes in the dataset was revised by the latest available gene information under the accession numbers of the respective genomes at NCBI. To this end, genes that are meanwhile marked as pseudo genes were removed from the dataset. No genes were added, since COG annotations of new genes are not available. We further omitted all genomes from subsequent analyses of which more than 10 pseudo genes were removed in this process. 93 genomes remained, comprising on average 2726 genes (minimum/ maximum number of genes: 784/8317).

*Gene family-free dataset*. Pairwise similarities between genes in the dataset were obtained using the relative reciprocal BLAST score (RRBS) [26]. Genes were compared on the basis of their encoding protein sequence using BLASTP+ [27] with an e-value threshold of 0.1 and disabled composition-based score adjustments.

For evaluation purposes, we produced different degrees of pruned gene similarity sets by filtering spurious gene similarities. For this, we employed an undirected variant of the stringency criterion (parameterized by *f *ϵ [0, 1]) for gene similarities proposed in [28], which is described in more detail in Additional file 1 Section 4.1.

To evaluate the gene family-free model, we ran an implementation of ACSI for *δ *= 0 on the unpruned gene similarity graph of our dataset and compared the 4015841 interval pairs with respect to the contained COG identifiers. We discarded all pairs for which at least one interval contained less than two genes with a COG identifier. In the remaining 1194036 interval pairs, we observed that the similarity in the set of COG identifiers depends strongly on the intervals' score (Table 1). Among the clusters with a score greater or equal 10, 95% have the same set of identifiers in both intervals. While this number decreases for smaller scores, still a quarter of the interval pairs with a score lower than 1 do not differ in their COG identifiers. This shows that our approach is able to detect gene clusters that would also be detected with well-established gene family based approaches.

**Table 1:** Statistics of overlaps between the COG identifier sets of pairs of weak common intervals.

						score					
	
overlap in %	*<*1	[1 *− *2[	[2 *− *3	[3 *− *4[	[4 *− *5[	[5 *− *6[	[6 *− *7[	[7 *− *8[	[8 *− *9[	[9 *− *10[	≥ 10
100	28.1	22.0	46.7	78.4	90.2	75.6	84.6	63.2	86.5	78.4	95.0
[80 *− *100[	0.0	0.0	0.1	0.2	0.4	1.8	2.0	10.4	8.2	18.5	4.9
[60 *− *80[	1.7	1.7	2.7	2.1	2.7	13.6	8.4	17.4	4.0	2.6	0.2
[40 *− *60[	12.0	14.7	18.5	9.9	2.4	2.5	2.1	5.0	0.7	0.3	0.0
[20 *− *40[	0.1	0.1	0.3	0.7	1.1	3.4	1.5	1.8	0.1	0.2	0.0
[0 *− *20[	58.1	61.4	31.8	8.8	3.2	3.1	1.4	2.7	0.6	0.2	0.0

Total	30002	239077	289450	253643	199372	49254	58889	17952	23603	4568	28226

Columns stand for bins of weak common interval scores, rows for bins of overlap sizes of the COG identifier sets of weak common interval pairs. Values are given in percent with respect to the total number of pairs per score bin given in the last row.

This is not a surprise, as weak common intervals are in fact a generalization of the classic common intervals model: A run of ACSI on a dataset where similarity scores are only set between members with the same COG identifiers finds the exact same set of clusters as the common intervals based approach.

To evaluate the predictive power of our approach, we compare the output of our program to gene clusters predicted by the *reference gene cluster algorithm *(RGC) [11]. While this algorithm is capable of multiple genome comparison and the detection of faint conservation patterns, we use it in this context for pairwise genome comparison to detect interval pairs (*I*_1_,*I*_2_) whose gene sets have a symmetric set distance of at most 2. It has been previously observed that the generalization to approximate conservation underlying the reference gene cluster approach is not only a way to find imperfectly conserved clusters, but also a means to add robustness against errors in gene family assignment. For example, an interval pair may appear to have a set distance of two because besides the shared genes, there is one gene unique to *I*_1 _and one gene unique to *I*_2_. However a closer inspection of the genes reveals that these genes are in fact homologs that were not recognized in the preceding partitioning of genes into homology families. We ran RGC on all pairs of the 93 genomes setting parameters *δ *= 2 (maximal tolerated symmetric set distance) and *s *= 3 (the minimum cluster size). The program returned among others 192900 "single-mismatch clusters", i.e. clusters that have exactly one extra gene in each interval. In 47453 (24.60%) of the single-mismatch clusters, we observe a similarity score between the two extra genes in our BLAST dataset. ACSI found 89.84% of the single-mismatch clusters and for 75.24% the extra genes turned out to be pairwise best hits. Moreover we observe that in 18143 among the single-mismatch gene clusters predicted by RGC the two extra genes have exactly the same annotation string. (Annotations containing the word "hypothetical" were ignored.) ACSI finds 90.19% of these clusters. Surprisingly, 4.59% of the single-mismatch clusters in which the two extra genes had best hits to each other were not found by ACSI. This is because for one or more of the other genes in the cluster our BLAST results did not return any similarity score to a gene in the other interval. Apparently the elements of a cluster of orthologous groups can be very faintly related in terms of sequence similarity.

*Comparison with RegulonDB data*. Among other information about transcriptional regulation, RegulonDB [29] provides a list of operon locations in *Escherichia coli *K12. While the majority of operons in RegulonDB are computationally predicted, some are also experimentally confirmed. From 2649 operons reported in RegulonDB, 846 span two or more genes. We mapped these operons to the annotation of the *E. coli *K12 genome in our data set. However, 104 operons contain genes that are not annotated in our dataset and thus were omitted from subsequent analysis. The remaining 742 operons span between 2 and 16 genes, 71.83% of which span 2 or 3 genes. The number of detected gene clusters depends strongly on the degree of evolutionary relatedness between the *E. coli *K12 genome and other genomes in the dataset. While ACSI and RGC predicted many occurrences in other close related *γ*-proteobacteria in our dataset, for the majority of genomes only few occurrences of operons were reported. Additional file 1 Section 4.2, gives an overview of the number of found gene clusters in the dataset. The sets of reported operons found by ACSI and RGC are not entirely overlapping. Instead, ACSI finds operons which RGC does not find and vice versa. A complete overview of unique findings for algorithms and parameter settings is shown in Table 2.

**Table 2:** Unique findings (with 100% overlap) of operons by ACSI and RGC with minimum cluster size *s *= 2 and varying parameters.

Unique to. . .	RGC	RGC	ACSI	ACSI	ACSI	ACSI
	*δ *= 0	*δ *= 2	*δ *= 0,	*δ *= 0,	*δ *= 2,	*δ *= 2,
			*f *= 0.0	*f *= 0.9	*f *= 0.0	*f *= 0.9
RGC *δ *= 0	-	118	133	119	190	175
RGC *δ *= 2	0	-	56	49	80	72
ACSI *δ *= 0, *f *= 0.0	4	45	-	0	61	52
ACSI *δ *= 0, *f *= 0.9	11	59	21	-	82	62
ACSI *δ *= 2, *f *= 0.0	0	8	0	0	-	0
ACSI *δ *= 2, *f *= 0.9	5	20	11	0	20	-

Each column shows the number of unique findings of an algorithm and parameter setting indicated by the column heading in comparison to algorithms and parameter settings specified in the rows.

## Conclusions

In this work we introduced a new model to detect gene clusters based on the study of (approximate) weak common intervals in indeterminate strings. In context of gene family-free analysis, we presented a scoring scheme for (approximate) weak common intervals which rates both interval size and the degree of similarity between the contained genes of an (approximate) weak common interval pair. We use our gene family-free model to predict gene clusters between pairs of genomes. This approach is evaluated in comparison with the common intervals-based reference gene cluster model.

In addition to the use case of detecting gene clusters, our algorithms can also be helpful to identify synteneous blocks in a gene family-free analysis. The hierarchical nature of common intervals is maintained in our weak common intervals model, which makes it ideal for studying potential synteneous blocks of arbitrary resolution. The basic concept of common intervals in strings has seen many generalizations in the past years which have greatly increased its utility for biological studies, in particular the simultaneous consideration of more than two strings, requiring common intervals to occur in all or at least a certain number of them. This generalization of (approximate) weak common intervals in indeterminate strings is undoubtedly an interesting direction for future work.

## Competing interests

The authors declare that they have no competing interests.

## Authors' contributions

All authors were involved in the early conception of the project. DD, KJ and JS developed the methods and designed the analysis. DD and KJ performed the evaluation and wrote the manuscript; all authors discussed the results, commented on the manuscript, and read and approved its final version.

## Supplementary Material

Additional file 1 Click here for file

## Declarations

We acknowledge support for the Article Processing Charge by the German Research Foundation and the Open Access Publication Fund of Bielefeld University Library.

This article has been published as part of *BMC Genomics *Volume 15 Supplement 6, 2014: Proceedings of the Twelfth Annual Research in Computational Molecular Biology (RECOMB) Satellite Workshop on Comparative Genomics. The full contents of the supplement are available online at http://www.biomedcentral.com/bmcgenomics/supplements/15/S6.
