# Supplementary material for: Identifying gene clusters by discovering common intervals in indeterminate strings
Source: BMC Genomics. 2014 Oct 17;15(Suppl 6):S2. doi: 10.1186/1471-2164-15-S6-S2 (PMC4274641; doi:10.1186/1471-2164-15-S6-S2)
Supplement: Additional file 1 [file 1471-2164-15-S6-S2-S1.pdf]

Supporting Information
for
Identifying Gene Clusters by Discovering Common Intervals in Indeterminate Strings

Daniel Doerr, Jens Stoye, Sebastian Böcker, and Katharina Jahn

August 6, 2014

1 Weak common intervals

1.1 Min-rank intervals

1.1.1 Computing min-rank intervals

Didier *et al.* [2] give a linear time algorithm to identify all rank intervals in a string, that we use to precompute all (min-) rank intervals for characters in T_S^i. Their bounds are stored in table $\textsc{Int}$ s.t. entry $\textsc{Int}[y]$ stores the bounds of the min-rank interval for character $T_S^i[y]$. Array $\textsc{Int}$ must be updated when proceeding from one level to the next, so that $\textsc{Int}[y]$, always points to the min-rank interval of the currently processed character in $T_S[y]$.

1.1.2 Updating array Int

Algorithm S1 updates array $\textsc{Int}$ for the next level. In the first part of the algorithm the min-rank interval of each position y containing the currently processed character j is copied to the position of its rank-nearest successor y' if such exists and if $\textsc{Int}[y'] \subset \textsc{Int}[y]$. Now, in the proof of Lemma 1 we showed that for any character j at any position y in T_S for which $T_S^i[y] \neq j$, there exists some $j' \leq j$ at some position y' such that $T_S^i[y'] = j'$ and the min-rank intervals of j and j' are identical. Let us for now assume that $j' < j$. Then j' is visited prior to j, therefore the min-rank interval of j' is passed from j' to a position containing $j'+1$, to $j'+2$, etc., eventually reaching character j on position y. Note that such a path must exist if $([i, j]_{I_S}, [k, l]_{T_S})$ are weak common intervals.

The case where $j' = j$ is treated in lines 7 onwards. To this end we iterate once through all positions $\textsc{Pos}[j+1]$ of the character $j+1$ that will be processed next. Whenever the algorithm finds a position y such that $T_S^i[y] = j+1$, previous and subsequent positions in $\textsc{Pos}[j+1]$ that reside in the corresponding min-rank interval are updated to $\textsc{Int}[y]$.

1.2 Computing Succ

In order to identify weak common interval pair $([i, j]_{I_S}, [k, l]_{T_S})$, the algorithm follows a path of rank-nearest successors from i to j within the bounds of min-rank interval $[k, l]_{T_S}$ of character j at a position y, $k \leq y \leq l$. By doing so, the algorithm avoids the costly computation of determining the character set in an interval, which would be otherwise necessary in verifying that all characters in $i, \ldots, j$ are contained in a min-rank interval $[k, l]_{T_S}$.

We can adopt Remark 3 from Didier *et al.* [2]:

Algorithm S1 Update algorithm for INT

Input: Arrays INT, POS, and SUCC, min-rank string T_S^i, and current level j
Output: Updated INT array for level $j+1$

// push rank intervals of j to successor positions
for each element y in POS[j] **do**
    $y' \leftarrow$ rank-nearest successor of y if exists, otherwise ∞
    if $y' \neq \infty$ **and** INT[y'] $\subset$ INT[y] **then**
        INT[y'] $\leftarrow$ INT[y]
    end if
end for
// Update rank intervals in the neighborhood of positions y for which $T_S^i[y] = j+1$
Initialize empty queue QUEUE
PREVIOUS $\leftarrow -\infty$
for each element y in POS[$j+1$] **do**
    if $T_S^i[y] \neq j+1$ **then**
        if PREVIOUS $\neq -\infty$ **and** $d^i_{T_S}(y, \text{PREVIOUS}) \leq j+1$ **then**
            INT[y] $\leftarrow$ INT[PREVIOUS]
        else
            Push y onto QUEUE
        end if
    else
        while QUEUE is not empty **do**
            Pop y' from QUEUE
            if $d^i_{T_S}(y, y') \leq j+1$ **then**
                INT[y'] $\leftarrow$ INT[y]
            end if
        end while
        PREVIOUS $\leftarrow y$
    end if
end for

Observation 1 *For each min-rank interval $[k,l]_{T_S}$ holds that the min-rank distance of any two positions inside the interval is strictly smaller than any min-rank distance of a position inside $[k,l]_{T_S}$ to a position outside the interval.*

Observation 1 ensures us that a path of rank-nearest successors from i to j along positions in T_S will always stay within bounds of min-rank interval $[k,l]_{T_S}$ of j for position y as long as $([i,j]_{I_S}, [k,l]_{T_S})$ are weak common intervals.

Let D_{T_S} be the table that stores indeterminate string T_S. In each iteration, the rank-nearest successors of all characters in T_S are precomputed and stored in a table SUCC, which is an analogous table to D_{T_S}. That is, the rank-nearest successor of character c at position y in T_S is stored at the same index position in SUCC as character c in D_{T_S}. Thus, the size of SUCC is $\mathcal{O}(\|T_S\|)$.

Since SUCC is traversed parallel to D_{T_S}, the look-up of the rank-nearest successor of a character c at position y in T_S can be performed in $\mathcal{O}(1)$ if one keeps track of the index position of c.

Constructing SUCC is achieved by sweeping through T_S once from left to right and once from right to left. Thereby most recent positions of observed characters $i, \ldots, n$ are tracked in an array OCC. Let p be the index position of a character $c \geq i$ at a position y in T_S in D_{T_S}, SUCC[p] is set to OCC[$c+1$] if

1. character $c+1$ has been observed in a previous position (including y) and

2. $\textsc{Succ}[p]$ is either undefined or $d^i_{T_S}(y, \textsc{Occ}[c+1]) < d^i_{T_S}(y, \textsc{Succ}[p])$ or $d^i_{T_S}(y, \textsc{Succ}[p]) = d^i_{T_S}(y, \textsc{Occ}[c+1])$ and $\textsc{Occ}[c+1] < \textsc{Succ}[p]$.

Min-rank distances can be computed in $\mathcal{O}(1)$ time after $\mathcal{O}(m)$ preprocessing by performing *range maximum queries* on T^i_S [1].

2 Precomputed data structures in the AWCII algorithm

The first speedup in the algorithm is accomplished by precomputing left and right bounds of all candidate intervals in T_S of the ith iteration, which are subsequently stored in tables L and R. To this end, we sweep for each $j \in [i, n]$ through all characters in T_S from one side to the other. Specifically, when sweeping from left to right, table R is constructed, table L in reverse direction. During each sweep we keep track of up to $\delta + 1$ outermost positions in T_S that contain any character in $[i, j]$ and are not more than $0, 1, \ldots \delta$ indels away from the current position. Whenever we come across character j at any position y, all tracked outermost positions and their corresponding number of indels are stored in L, respectively R. Precomputation of L and R in the ith iteration takes $\mathcal{O}((\delta + 1) \cdot n\|T_S\|)$ time and requires $\mathcal{O}((\delta + 1) \cdot \|T_S\|)$ space.

The next improvement in performance of the algorithm is achieved by precomputing table $\textsc{RangeContent}$, which stores for each candidate interval $[k, l]_{T_S}$ corresponding to character j its character content shared with $[i, j]_{I_S}$, i.e. $C = \mathcal{C}(T_S[k, l]) \cap [i, j]$. The size of C is required in line 16 in Algorithm AWCII to compute δ_S. Consequently we store for each interval corresponding to character j at position y in T_S and defined by bounds in L and R, the number of characters that are within $[i, j]$. In other words, $\textsc{RangeContent}$ stores at most $(\delta + 1)^2$ entries associated with candidate intervals of character j at position y in T_S.

Computing $\textsc{RangeContent}$ can be achieved by sweeping again for each $j \in [i, n]$ once from left to right and once from right to left through all characters in T_S. At first, table $\textsc{RangeContent}$ is initialized with zeros. Every entry in $\textsc{RangeContent}$ serves as counter for the number of characters that are within $[i, j]$ of a candidate interval in T_S. In each sweep we keep track of the most recent position p_j in which character j has been observed. We process every character c of each position in T_S for which $i \leq c \leq j$. Going through all combinations (k, l) of interval bounds in L and R for character c, we increase the counter of $[k, l]_{T_S}$ as long as $p_j \in [k, l]_{T_S}$ and the counter has not yet been increased for character j. The latter condition prevents that a character is counted twice, once from a sweep from left to right and then again when sweeping from right to left.

3 Performance improvements of ACSI

We now describe the runtime heuristic improvements that were made in ACSI (see Algorithm ACSI in main article). For a broad description of the algorithm, see Section *A runtime heuristic for discovering approximate weak common intervals* in the main article. Instead of iterating through all $j \in [i, n]$ in lines 4-6, a heuristic improvement is achieved by choosing only those values $\mathcal{J} \subseteq [i, n]$ that are reachable within at most δ indels around position y in T_S. To this end, the algorithm finds the leftmost position $k' <= y$ in T_S such that $|\{p \mid k' \leq p \leq y : T_S[p] \cap [i, n] = \emptyset\}| \leq \delta$. Likewise it identifies the rightmost position $l' \geq y$ such that $|\{p \mid y \leq p \leq l' : T_S[p] \cap [i, n] = \emptyset\}| \leq \delta$. We can now set $\mathcal{J} = \mathcal{C}(T_S[k', l']) \cap [i, n]$. In practice $|\mathcal{J}| \ll n - i + 1$. Identifying k', l' and $\mathcal{J}$ is achievable within $\mathcal{O}(n + m)$ time and $\mathcal{O}(n)$ space. The algorithm further improves in runtime in practice by identifying the largest j' such that $|\mathcal{J} \cap [i, j']| \leq \delta$. We then reiterate the process of identifying the leftmost position k' such that $|\{p \mid k' \leq p \leq y : T_S[p] \cap [i, j'] = \emptyset\}| \leq \delta$. Likewise we also determine the new leftmost position l'' and set $\mathcal{J} = \mathcal{C}(T_S[k'', l'']) \cap [i, j']$. This process is iterated until the set $\mathcal{J}$ no longer changes.

Stringency f	Avg. #singleton genes	Avg. pairwise similarities	Avg. connectedness
unpruned	3262	6499	1.93
0.1	3271	6242	1.85
0.2	3312	5349	1.60
0.3	3364	4137	1.25
0.4	3420	3095	0.96
0.5	3486	2336	0.74
0.6	3565	1784	0.58
0.7	3661	1384	0.46
0.8	3775	1088	0.38
0.9	3901	877	0.31

Table S1: Average number of singleton genes, average number of pairwise similarities, as well as the average connectedness of a gene as a function of stringency in our dataset. The average number of genes per pairwise comparison in our dataset is 5444. Singleton genes are genes that are not similar to any other gene in a pairwise comparison. The connectedness of a gene in a pairwise comparison is the number of other genes it is similar to.

4 Supporting information of the evaluation

4.1 Stringency filter

Given chromosomal strings A and B, the stringency filter takes into account gene similarities incident to genes $x \in A$ and $y \in B$, when computing a local threshold value for gene similarity $\sigma_{AB}(x, y)$. The maximal similarity of a gene x in A is given by $\sigma_{AB}^{\max}(x) = \max_{y' \in B}(\sigma_{AB}(x, y'))$. Likewise for a gene y in B, $\sigma_{AB}^{\max}(y) = \max_{x' \in A}(\sigma_{AB}(x', y))$. The *stringency* parameter $f \in [0, 1]$ allows to restrict the set of gene similarities to those with a certain degree of locally minimal weight:

$$\sigma_{AB}(f, x, y) = \begin{cases} \sigma_{AB}(x, y) & \text{if } \sigma_{AB}(x, y) \geq f \cdot \max(\sigma_{AB}^{\max}(x), \sigma_{AB}^{\max}(y)) \\ 0 \quad \text{otherwise.} \end{cases}$$

The effects in the choice of parameter values for f captured on 3 different properties of our dataset in pairwise comparisons are shown in Table 4.1.

4.2 Comparison with RegulonDB

Figure S1 gives an overview of the number of found gene clusters in the dataset, in which genomes are ordered according to the cumulative sum of operon findings by ACSI. We differentiated between findings, where the gene set of a predicted gene cluster was a superset of genes of an operon (labeled "100% overlap"), more than 50%, or at most 50% of genes overlapped. In cases where multiple gene clusters in different locations of the other genomes contained operon genes, we chose the prediction with the highest overlap. The graph visualizing the sum over all 100%, $> 50\%$, and $\leq 50\%$ overlaps is labeled as "cumulative". Out of the 742 operons, 633 are entirely overlapped by gene clusters reported by ACSI in all genome comparisons against *E. coli* K12 in the unpruned graph and 612 operons for a pruned graph with $f = 0.9$. In comparison, RGC with $\delta = 0$ and $s = 2$ finds 504 operons, whereas 622 operons are found with parameter settings $\delta = 2$ and $s = 3$.

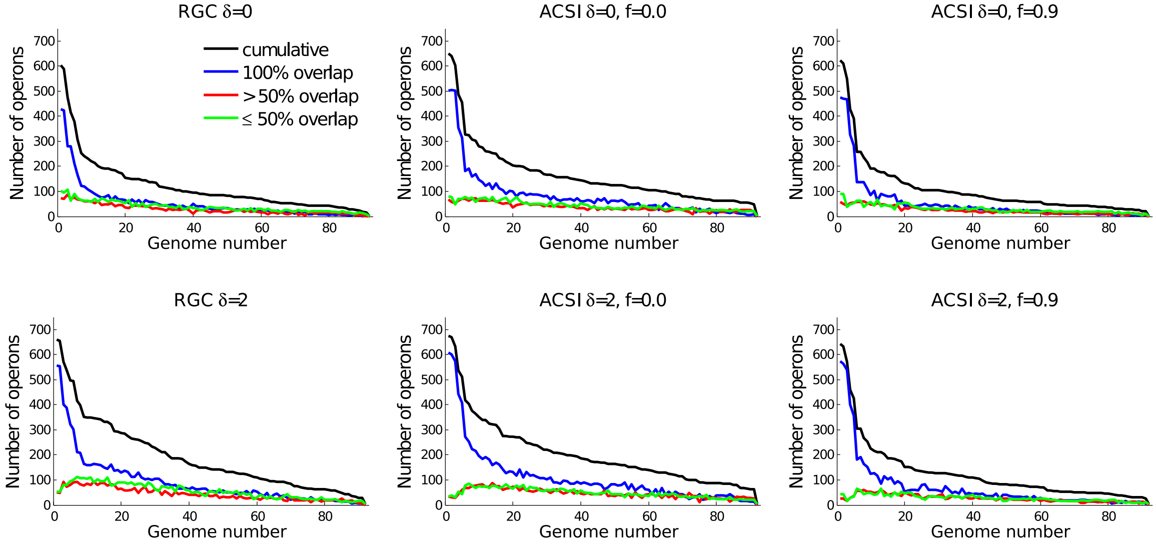

Figure S1: Number of operons in genomes reported by RGC with parameter settings $\delta = 0$, $s = 2$ (top left) and $\delta = 2$, $s = 3$ (bottom left) and ACSI in unpruned graphs (middle) and pruned graphs with $f = 0.9$ (right) for $\delta = 0$ (top) and $\delta = 2$ (bottom). Genomes are ordered according to the cumulative sum of reported operons.

References

[1] Michael A. Bender and Martn Farach-Colton. The lca problem revisited. In GastonH. Gonnet and Alfredo Viola, editors, *Proc. of LATIN 2000*, volume 1776 of *Lecture Notes in Computer Science*, pages 88–94. Springer Berlin Heidelberg, 2000.

[2] Gilles Didier, Thomas Schmidt, Jens Stoye, and Dekel Tsur. Character sets of strings. *J. Discr. Alg.*, 5(2):330–340, 2007.
